# RiceNet v2: an improved network prioritization server for rice genes

**DOI:** 10.1093/nar/gkv253

**Published:** 2015-03-26

**Authors:** Tak Lee, Taeyun Oh, Sunmo Yang, Junha Shin, Sohyun Hwang, Chan Yeong Kim, Hyojin Kim, Hongseok Shim, Jung Eun Shim, Pamela C. Ronald, Insuk Lee

**Affiliations:** 1 Department of Biotechnology, College of Life Sciences and Biotechnology, Yonsei University, Seoul, Korea; 2 The Joint Bioenergy Institute, Emeryville CA and Department of Plant Pathology and the Genome Center, University of California, Davis, CA 95616, USA

## Abstract

Rice is the most important staple food crop and a model grass for studies of bioenergy crops. We previously published a genome-scale functional network server called RiceNet, constructed by integrating diverse genomics data and demonstrated the use of the network in genetic dissection of rice biotic stress responses and its usefulness for other grass species. Since the initial construction of the network, there has been a significant increase in the amount of publicly available rice genomics data. Here, we present an updated network prioritization server for *Oryza sativa* ssp. *japonica*, RiceNet v2 (http://www.inetbio.org/ricenet), which provides a network of 25 765 genes (70.1% of the coding genome) and 1 775 000 co-functional links. Ricenet v2 also provides two complementary methods for network prioritization based on: (i) network direct neighborhood and (ii) context-associated hubs. RiceNet v2 can use genes of the related subspecies *O. sativa* ssp. *indica* and the reference plant *Arabidopsis* for versatility in generating hypotheses. We demonstrate that RiceNet v2 effectively identifies candidate genes involved in rice root/shoot development and defense responses, demonstrating its usefulness for the grass research community.

## INTRODUCTION

Rice is a most widely consumed staple food crop. Rice not only serves as a major food source, but is also an excellent model for the study of other monocotyledonous plant species including many cereals and bioenergy crops due to its desirable attributes as a model crop: compact genome size, well annotated genome, abundant functional genomics data and well-established methods for genetic transformation.

Although rice is the first crop to have its genome completely sequenced (1), knowledge about gene-to-trait association is still scarce (2). Forward genetics approaches, map-based cloning strategies and other methods have led to the identification of genes encoding important rice traits. However, most of the genetic components underlying rice traits are still unknown. Recent advances in whole genome microarrays of rice have accumulated vast amount of functional genomics data related to the important traits (3). More recently, resequencing-based population genetics studies have generated an unprecedented amount of data on genetic variants, associated with important traits (4).

Genes that contribute to the important phenotypes often function as a group rather than individually. This modular or pathway nature of biological processes provides an opportunity of identifying trait-associated genes based on guilt-by-association principle (5,6). Functional gene networks have been demonstrated to serve as powerful approach for generating holistic models of pathways in many organisms including plants (2,7). We previously constructed a genome-scale functional network server for rice, called RiceNet, and demonstrated its usefulness in identifying genes that are involved in biotic stress responses (8). With the remarkable growth of the availability of rice genomics data since the initial construction of RiceNet, we have updated the network and generated an effective web-based network analysis server to improve genetic dissection of rice traits.

In this paper, we present an improved network prioritization server for *Oryza sativa* ssp. *japonica* genes, RiceNet v2 (http://www.inetbio.org/ricenet), in which substantially larger amount of data, improved machine learning algorithms and network analysis methods were incorporated. RiceNet v2 increases the coverage of genome and the number of co-functional links, potentially improving prediction power for trait-associated genes. RiceNet v2 also includes two complementary network prioritization algorithms. In addition to *O. sativa* ssp. *japonica* genes, the web-server can use *O. sativa* ssp. *indica* genes and *Arabidopsis* genes, enabling researchers to use prior knowledge derived from a related subspecies or a reference model plant to guide search of novel candidate genes in the network. This enhanced network and gene prioritization method will facilitate effective hypothesis generation about the function of the estimated 37K rice genes.

## NETWORK CONSTRUCTION

RiceNet v2 was constructed by machine learning of diverse types of large-scale genomics data. Detailed description of network construction methods can be found in Supplementary Online Methods. Component genes of RiceNet v2 were derived from 36 736 *O. sativa* ssp. *japonica* non-TE element protein coding genes annotated by Os-Nipponbare-Reference-IRGSP-1.0 (9). Twenty-one component networks inferred from different data types are summarized in Table 1. Comparisons between RiceNet v2 and the previous network, RiceNet v1, in terms of data sources and analysis methods for component networks are summarized in Supplementary Table S1. In summary, the major differences from the previous network are (i) improved algorithms to infer co-functional links from gene neighborhood (10), (ii) new associalogs from the latest networks for other species (7,11,12), (iii) substantially larger amount of expression data derived from Gene Expression Omnibus (GEO) database (13) for co-expression links and (iv) associalogs of new co-expression networks for human, fly (*Drosophila melanogaster*) and zebrafish (*Danio rerio*). The gold standard co-functional gene pair used for network training was generated by pairing *O. sativa* ssp. *japonica* genes that share the same pathway annotations by at least one of the following four databases: (i) KEGG (Kyoto Encyclopedia of Genes and Genomes) (14), (ii) Gene Ontology biological process (GO-BP) annotated by Biofuel Feedstock Genomics Resource (BFGR) (15), (iii) MapMan (16) and (iv) RiceCyc (17). Genes annotated for broad pathway concepts were excluded from gold standard gene pairs (see Supplementary Online Methods), because they generate excessive number of gene pairs for the pathways, potentially leading to biased training (18). These processes generated a gold standard set of 591 664 positive and 58 416 152 negative gene pairs. Bayesian statistics framework measured likelihood of two paired genes to participate in the same pathway using the gold standard pathway gene pairs as for the previous network (8). The 21 component networks with log likelihood scores were then integrated into a single network by the weighted sum method described for RiceNet v1 (8). The final integrated RiceNet v2 contains 25 765 genes (70.1% of coding genome) connected by 1 775 000 co-functional links. Edge information of the integrated RiceNet v2 and all of the 21 component networks is available from the web-sever download page of http://www.inetbio.org/ricenet/.

**Table 1.** Summary of 21 component networks and RiceNet v2

(Network Code) Description	# Genes (coding genome coverage,%)	# Links
(AT-CC) Co-citation of *Arabidopsis thaliana* orthologs among full-text articles from PubMed Central	4492 (12.2)	65 497
(AT-CX) Co-expression of *A. thaliana* orthologs across microarray experiments	11 309 (30.8)	426 000
(AT-HT) Protein-protein interactions between *A. thaliana* orthologs measured by high-throughput experiments.	2075 (5.6)	6715
(AT-LC) Protein-protein interactions between *A. thaliana* orthologs from literature	1043 (2.8)	2951
(CE-CC) Co-citation of *Caenorhabditis elegans* orthologs among full-text articles from PubMed Central	3011 (8.2)	102 000
(CE-CX) Co-expression of *C. elegans* orthologs across microarray experiments	3923 (10.7)	100 000
(DM-CX) Co-expression of *Drosophila melanogaster* orthologs across microarray experiments	4338 (11.8)	166 000
(DM-HT) Protein-protein interactions between *D. melanogaster* orthologs measured by high-throughput experiments.	2932 (8.0)	12 000
(DR-CX) Co-expression of *Danio rerio* orthologs across microarray experiments	4574 (12.5)	159 000
(HS-CX) Co-expression of *Homo sapiens* orthologs across microarray experiments	2772 (7.5)	50 349
(HS-HT) Protein-protein interactions between *H. sapiens* orthologs measured by high-throughput experiments	2864 (7.8)	30 000
(HS-LC) Protein-protein interactions between *H. sapiens* orthologs from literature	4300 (11.7)	73 000
(OS-CX) Co-expression of *O. sativa* genes across microarray experiments	21 745 (59.2)	597 180
(OS-GN) Genomic neighborhood of *O. sativa* orthologs among prokaryotic genomes	5259 (14.3)	246 000
(OS-LC) Protein-protein interactions between *O. sativa* genes from literature	103 (0.3)	172
(OS-PG) Phylogenetic profile similarity between *O. sativa* genes	2218 (6.0)	38 000
(SC-CC) Co-citation of *Saccharomyces cerevisiae* orthologs among MEDLINE abstracts	3890 (10.6)	91 000
(SC-CX) Co-expression of *S. cerevisiae* orthologs across microarray experiments	3819 (10.4)	204 000
(SC-GT) Similarity of genetic interactions between *S. cerevisiae* orthologs	3449 (9.4)	136 000
(SC-HT) Protein-protein interactions between *S. cerevisiae* orthologs measured by high-throughput experiments	4318 (11.8)	273 000
(SC-LC) Protein-protein interactions between *S. cerevisiae* orthologs from literature	4121 (11.2)	113 000
(RiceNet v2) full integrated network	25 765 (70.1)	1 775 000

## NETWORK ASSESSMENT

RiceNet v2 covers 7388 more rice genes and maps ∼1.2 million more co-functional links than RiceNet v1. If network accuracy has been maintained or augmented, an increase in network information is expected to improve the prediction power of the network. To access network accuracy, we used the GO-BP annotations from the agriGO database (19), which is independent from both RiceNet v2 and RiceNet v1 training data. We excluded the top 20 largest GO-BP terms to avoid biased assessment towards those terms, resulting in a total of 267 187 positive gene pairs and 16 549 913 negative gene pairs for network validation. The gene pairs for validation have ∼11% and ∼6% overlap with gold standard positive gene pairs for training RiceNet v2 and RiceNet v1, respectively, confirming independence of validation data from the original training data. We assessed network performance using precision-recall analysis, where network precision for the given genome coverage is measured by odds ratio (OR):

\begin{equation*} \begin{array}{*{20}l} {{\rm OR} = } {\frac{{({\rm \# positive\;gene\;pairs\;in\;network})\;/\;({\rm \# negative\;gene\;pairs\;in\;network})}}{{{\rm \# total\;positive\;gene\;pairs\;/\;\# total\;negative\;gene\;pairs}}}} \\ \end{array} \end{equation*}

**Figure 1.**
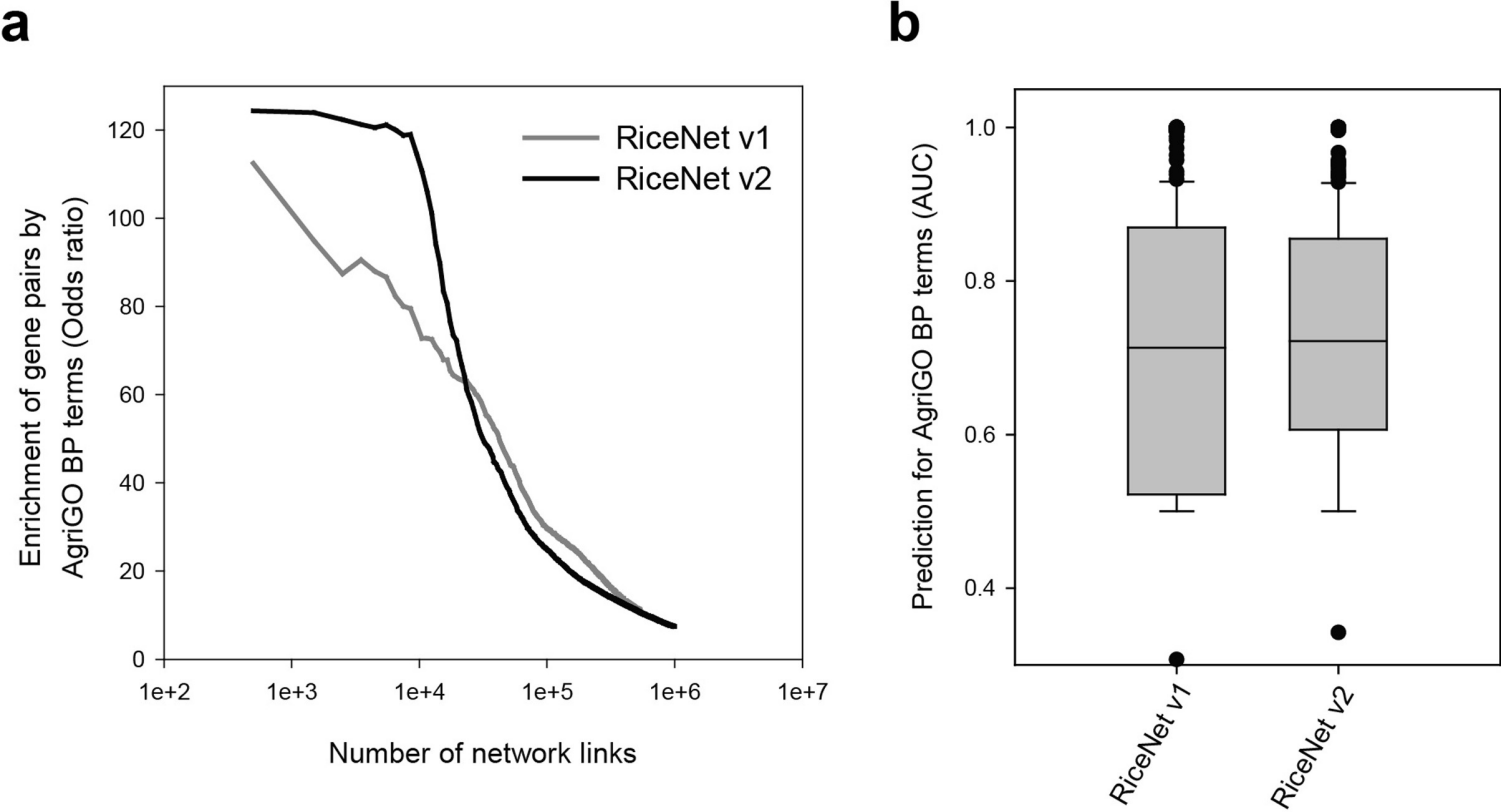
Network assessment using a set of validation gene pairs based on agriGO biological process (BP) annotations. (**a**) Precision-recall analysis to compare RiceNet v1 and RiceNet v2. The precision of co-functional links were measured by odds ratio (OR). RiceNet v2 shows substantially higher OR than RiceNet v1 for high scored network links. (**b**) A box-and-whisker plot of network prediction powers for 336 agriGO BP terms with more than four annotated genes, measured by area under the curve from ROC analysis. RiceNet v2 shows significantly higher prediction power for the processes than RiceNet v1 (*P* = 1.11 × 10^-6^, Wilcoxon signed rank test). To avoid network size effect on prediction power, we used only top 588 221 links of RiceNet v2 to match the size of RiceNet v1 during analysis.

We find RiceNet v2 shows substantially higher OR among higher ranked gene pairs than RiceNet v1 (Figure 1a).

The improved accuracy for the high scoring network links of RiceNet v2 is expected to improve functional predictions based on guilt-by-association. To test this, we measured network prediction power based on receiver operating characteristic (ROC) analysis, which can be summarized into an area under curve (AUC) score. In this analysis setting, we prioritize all genes of the network by direct connections to the known genes for a phenotype, called *guide genes*. If the network is predictive for a phenotype, known phenotype genes might be modular and the member genes would be highly ranked by high interconnectivity, resulting in a high AUC score. From analyzing prediction power for 336 GO-BP terms by agriGO annotations, we observed significant increase in AUC score distribution by RiceNet v2 (*P* = 1.11 × 10^−6^, Wilcoxon signed rank test) (Figure 1b).

## A WEB SERVER FOR GENE PRIORITIZATION

**Figure 2.**
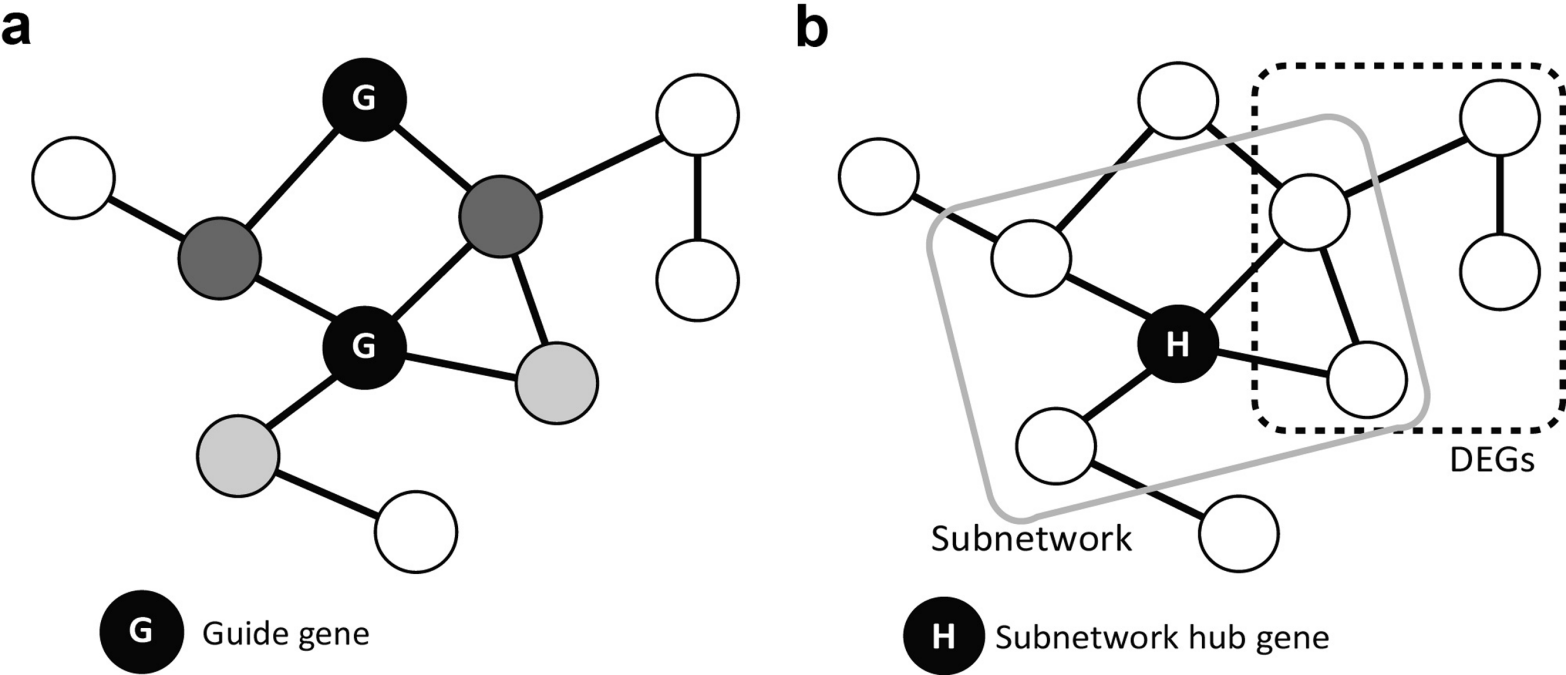
Schematic figures of two complementary network prioritization methods included in RiceNet v2. (**a**) Gene prioritization based on network direct neighborhood. Candidate genes are ranked by total connection score (sum of edge weight scores) to the all direct neighbors of guide genes (black node marked by G), which are already known to be involved in a query phenotype. Darker gray nodes represent more likely candidate genes. (**b**) Gene prioritization based on context associated hub. Each predefined subnetwork (enclosed by the gray rounded rectangle) comprises a central hub (black node marked by H) and its connected neighbor genes. If the subnetwork neighbor genes significantly overlap with DEGs from a query phenotype context (enclosed by the dotted rounded rectangle), the subnetwork hub gene is assigned as a context-associated hub, which is a new candidate for the phenotype.

Network-assisted gene prioritization for phenotypes of interest has proven effective in tackling genetic dissection of complex traits in many organisms (20). The previous RiceNet web server provided only a single method of gene prioritization, based on network direct neighborhood analysis. RiceNet v2 provides two complementary methods of network-based gene prioritizations (Figure 2), which better utilizes network information from publicly available rice gene-to-phenotype association mapping (described below). In addition, the new web server can accept input guide genes from *Arabidopsis* and the rice subspecies, *O. sativa* ssp. *indica*. This feature allows RiceNet v2 to harness ‘homologous guide genes’ from the most intensively studied model plant, *Arabidopsis*, as well as the other major rice subspecies.

### Gene prioritization based on network direct neighborhood

In this option, novel genes for a particular phenotype are prioritized by strength of their direct connection to known genes governing the phenotype, namely ‘guide genes’ (Figure 2a). In this case, only direct neighborhoods of guide genes are considered as new candidates. RiceNet v2 uses the sum of edge weight scores (i.e. sum of log likelihood scores) of all direct connections to the guide genes for prioritization, and lists the top 100 novel candidate genes. A full list of candidate genes with ranks and other relevant information is downloadable as a text file. We also provide paralogs of candidate genes derived from Plant Genome Duplication Database (PGDD) (21) to inform users about potential functional back-up effect during mutant phenotype assay.

### Gene prioritization by context associated hubs

For many rice traits, known associated genes (i.e. guide genes) are scarce, due to the technical difficulties in functional validation. The lack of guide genes is therefore currently the biggest hurdle in efficiently utilizing the network, which prioritizes genes based on guilt-by-association principle. An alternative approach to study gene-to-phenotype association is use of genes that show altered expression in a phenotype-relevant context. For example, biotic or abiotic stress changes expression of many rice genes. Identification of differentially expressed genes (DEGs) between control and trait-relevant conditions is relatively easy, and we already have trait-associated DEGs for many rice traits in public databases. Genome-wide expression profiling upon stress condition may elucidate some stress response regulators among DEGs. However, many of the DEGs are simply a consequence of an altered cell state upon stress response rather than actual stress response regulator. In addition, many regulators do modulate other genes without changing their expression levels significantly upon stress condition. RiceNet v2 can prioritize genes for stress response using DEGs from specific stress conditions. Assuming regulators are functionally associated with many target genes, we first selected 13 174 genes with more than 50 connected neighbors by RiceNet v2. Then, we defined subnetworks for each of the 13 174 genes and their neighbors. If a central hub gene of a subnetwork modulates stress response, many of its subnetwork neighbors could be also DEGs. Hence, we measured statistical overlap between DEGs and neighbors of each selected subnetwork. The hub genes having neighbors that significantly overlap with DEGs are dubbed *context-associated hubs* (Figure 2b), which could be novel candidate genes for stress response.

### Use of functional information derived from *Arabidopsis* or a related subspecies of rice

RiceNet v2 can use genes of *O. sativa* ssp. *indica* and *Arabidopsis*. Users can submit guide genes using the Rice Information System (RIS) (22) gene ids. *O. sativa* ssp. *japonica* orthologs of *O. sativa* ssp. *indica* genes are pre-mapped by BLASTp (23) bidirectional best hits. Because the genomes of the two rice subspecies are highly conserved, we adopted a strict orthology threshold (*E*-value ≤ 1 × 10^-4^ for both directional hits) to avoid spurious orthologous relationships. Network prioritization is carried out for only homologous genes between two subspecies. The RiceNet v2 web server also accepts *Arabidopsis* genes. Orthology mapping between *O. sativa* ssp. *japonica* and *Arabidopsis* genes is less stringent, including inparalogs (24). The *orthologous guide genes* enables the user to benefit from the extensive functional information for both rice subspecies and from *Arabidopsis*.

## CASE STUDIES

**Figure 3.**
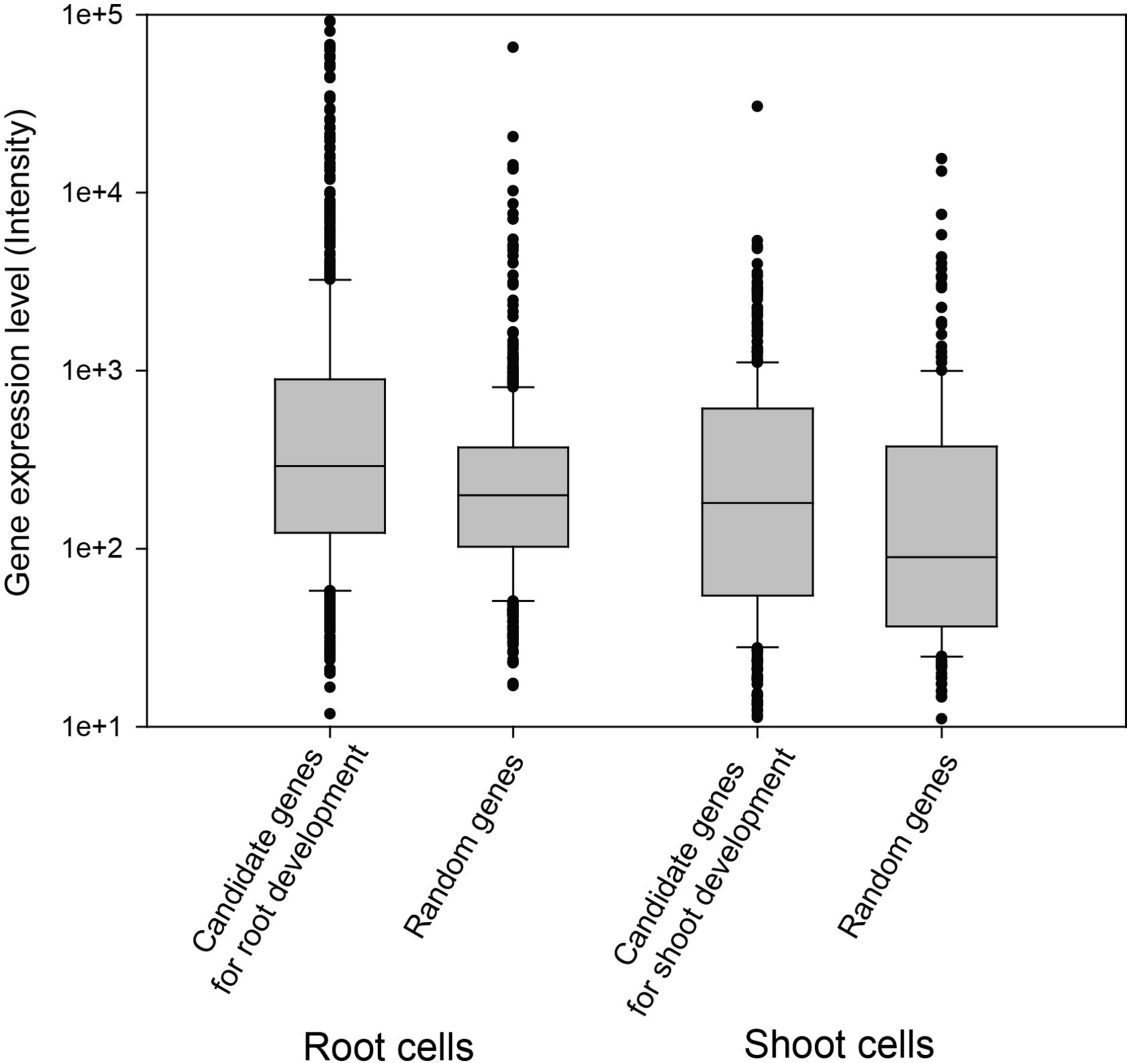
Validation of new candidate rice genes for root or shoot development based on network direct neighborhood method. Due to the lack of known rice genes for the traits, 60 and 19 *Arabidopsis* genes known for root and shoot development were submitted as guide genes, respectively. We validated new candidate genes using tissue specific expression atlas data, assuming genes for root development more actively express in root and genes for shoot development more actively express in shoot. We observed that top 100 candidate genes by RiceNet v2 show significantly higher expression levels than random genes in both target tissues: *P* = 1.3 × 10^-12^ (Wilcoxon rank sum test) for expression of root development gene candidates in root cells (left two box plots) and *P* = 7.2 × 10^-4^ (Wilcoxon rank sum test) for expression of shoot development gene candidates in shoot cells (right two box plots).

As described above, rice genes annotated with experimental evidences are scarce. For example, as of January 2015, there are only eight rice genes annotated for root development by Gramene GO-BP with a GO evidence code of IMP (inferred from mutant phenotype), while 60 *Arabidopsis* genes are annotated for root development with IMP. Similarly, only one rice gene is annotated, but 19 *Arabidopsis* genes are annotated for shoot system development by GO-BP with IMP. Therefore, it is a useful strategy to prioritize novel rice genes for root or shoot development using *Arabidopsis* orthologs for the equivalent traits. The likelihood of the new candidates could be validated by tissue-specific expression data. This approach assumes that genes for root development are expressed more actively in root cells and that genes for shoot development are more actively expressed in shoots. To test this approach we submitted 60 *Arabidopsis* genes demonstrated to control root development to the RiceNet v2 server, which returned 6012 new candidate rice genes for the phenotype. For validation, we employed a transcriptome atlas of rice cell types (25) (GEO accession: GSE13161), which provides expression profiles for 40 distinct cell types from rice shoot, root and germinating seed at several developmental stages. We compared expression levels of the top 100 candidates and 100 random genes, and observed significantly higher expression levels of top candidates from root cells (*P* = 1.3 × 10^-12^, Wilcoxon rank sum test) (Figure 3, left). We performed a similar analysis for shoot system development using the 19 *Arabidopsis* genes known to be involved in shoot system development as guide genes. RiceNet v2 server returned 2680 new rice candidate genes for the shoot system development. From comparison of expression levels between top 100 candidates and 100 random genes, we observed that top candidates show significantly higher expression levels than random ones in shoot cells (*P* = 7.2 × 10^-4^, Wilcoxon rank sum test) (Figure 3, right).

To test effectiveness of the prediction based on *context-associated hubs*, we prioritized genes for stress responses. We submitted 189 DEGs (*P* ≤ 0.01) for Xa21 mediated immune response (GEO accession: GSE22112) (26) to the RiceNet v2 web server and identified 183 context-associated hubs (*P* ≤ 0.01, Fisher's exact test) as new candidate genes. To validate the predictions, we measured enrichment of 834 annotated genes by two Gramene (17) GO-BP terms related to defense response—defense response, defense response to bacterium—among predicted 183 genes, and found significant enrichment of the annotated defense response genes among the new candidates (*P =* 1.09 × 10^-6^, Fisher's exact test). Conversely, we did not observe significant enrichment of defense response genes among 189 DEGs (*P =* 0.81). Notably, the top 183 hub genes of RiceNet v2 did not show any overlap with the 834 annotated defense response genes, indicating that the observed prediction power did not stem from intrinsic information of network structure. Users can reproduce these case studies by submitting example gene sets to RiceNet v2 web server.

## CONCLUSIONS

RiceNet v2 is an updated network prioritization web-server for rice. When compared with the previously published network server, RiceNet v1, RiceNet v2 is substantially improved in terms of both genome coverage and network accuracy, leading to enhancement in prediction power. RiceNet v2 provides two complementary network prioritization algorithms based on: (i) network direct neighborhood and (ii) context-associated hubs, facilitating efficient generation of testable hypothesis. In addition, RiceNet v2 now accepts guide genes from both *O. sativa* ssp. *indica* and *Arabidopsis* genes. This element allows user to build on the extensive prior knowledge of these genomes. We will continue to improve the rice gene network by incorporating sequencing-based gene expression data, the amount of which will rapidly grow in the coming years (27).

## SUPPLEMENTARY DATA

Supplementary Data are available at NAR Online.

SUPPLEMENTARY DATA
