# Supplementary material for: RiceNet v2: an improved network prioritization server for rice genes
Source: Nucleic Acids Res. 2015 Mar 26;43(Web Server issue):W122–7. doi: 10.1093/nar/gkv253 (PMC4489288; doi:10.1093/nar/gkv253)
Supplement: SUPPLEMENTARY DATA [file supp_43_W1_W122__index.html]

RiceNet v2: an improved network prioritization server for rice genes — RiceNet v2: an improved network prioritization server for rice genes — SUPPLEMENTARY DATA 

# RiceNet v2: an improved network prioritization server for rice genes

## SUPPLEMENTARY DATA

**Files in this Data Supplement:**

- SUPPLEMENTARY DATA
