# Supplementary material for: RiceNet v2: an improved network prioritization server for rice genes
Source: Nucleic Acids Res. 2015 Mar 26;43(Web Server issue):W122–7. doi: 10.1093/nar/gkv253 (PMC4489288; doi:10.1093/nar/gkv253)
Supplement: SUPPLEMENTARY DATA [file supp_gkv253_nar-00302-web-b-2015-File005.pdf]

RiceNet v2: an improved network prioritization server for rice genes

Tak Lee, Taeyun Oh, Sunmo Yang, Junha Shin, Sohyun Hwang, Chan Yeong Kim, Hyojin Kim, Hongseok Shim, Jung Eun Shim, Pamela C. Ronald, and Insuk Lee

Supplementary Online Methods

Defining a gene set for RiceNet v2

From 39,054 Non-TE locus from Os-Nipponbare-Reference-IRGSP-1.0 (1), we excluded 2,619 hypothetical proteins from the genes for RiceNet v2. We also excluded ChrSy and ChrUn genes because these genes were not mapped to any chromosomes. Including mitochondrial and chloroplast genes, total 36,736 genes were considered in constructing RiceNet v2. If either of two genes of a network link does not belong to these genes, the link was removed.

Gold standard gene pairs for training RiceNet v2

The Gene Ontology biological process (GO-BP) terms annotated by Biofuel Feedstock Genomics Resource (2), Kyoto Encyclopedia of Genes and Genomes (KEGG) metabolic pathways (3), MapMan metabolic pathways (4) and known/predicted biochemical pathways from RiceCyc (5) have been used to generate gold standard gene pairs to train the networks. The gold standard gene pairs were generated by pairing all the genes in each annotated terms. This method can give rise to training bias if a term has too many annotated genes because there will be too many gold standard gene pairs from a single term which may cause functional bias towards those terms. To minimize the training bias, ten biggest GO-BP terms were ignored during gold standard set construction. The gold standard set from GO-BP annotations was composed of 75,732 positive gene pairs and 4,937,629 negative gene pairs covering 3,167 *O. sativa* genes, ~9% of the 36,736 genes for network construction. For the same purpose of bias reduction during construction of a gold standard set based on KEGG, we ignored two biggest terms and five broad-concept terms of the KEGG pathways (release 72.0). Excluding the seven metabolic pathway terms resulted in a gold standard set of 290,809 positive and 8,384,886 negative gene pairs for 4,166 *O. sativa* genes, ~11% of the 36,736 genes. To generate gold standard from MapMan metabolic pathways, we generated gene pairs from pathways of third or fourth subBINs of hierarchy, because first and second BIN contains broad concept terms. We also ignored all the BINs starting with 35 because they are unknown. We ignored 11 pathways with vast number of annotated genes during gene pairing, resulted in a gold standard set of 201,359 positive and 22,29,3919 negative gene pairs for 6,708 genes, ~18% of the 36,736 genes. Lastly, for RiceCyc (version 3.3), we generated the gold standard pairs with ignoring three biggest pathways, resulted in 90,014 positive and 3,001,327 negative gene pairs for 2,487 genes, ~7% of the 36,736 genes. We combined all of the four sets of gold standard gene pairs to generate the integrated gold standard set, composing 591,664 positive and 58,416,152 negative gene pairs for 10,864 *O. sativa* genes, ~30% of the 36,736 genes. The excluded pathway terms during gold standard construction are listed at **Supplementary table 2**.

Benchmarking and integrating inferred functional links

Functional associations between genes from experimental, computational data were inferred by calculating the likelihood ratio (Log likelihood score, *LLS*) based on Bayesian statistics framework. *LLS* was calculated with the following equation;

$$\mathrm{LLS} = \ln\left(\frac{P(L|D)/P(\neg L|D)}{P(L)/P(\neg L)}\right)$$

where $P(L|D)/P(\neg L|D)$ is the odds of gold standard positives ($P(L|D)$) and negatives ($P(\neg L|D)$) for a given data. $P(L)/P(\neg L)$ is the odds of all gold standard positives ($P(L)$) and negatives ($P(\neg L)$). A network functional link can be supported by many multiple data types with different *LLS*s. Since not all of the data for integration are fully independent, naïve Bayesian integration is not a plausible approach. Hence, we used the weighted sum (*WS*) formula to integrate the data by modifying naïve Bayesian (6). The *WS* is defined as

$$WS = S_0 + \sum_{i=1}^{n} \frac{S_i}{D \times i}\ , for\ all\ S\ \geq T,$$

where S is the *LLS*. S_0 is the best *LLS*s and S_i is *LLS* of *i*th rank. D is a free parameter that is used to give weight. T is the minimum *LLS* threshold. Weighted sum takes full score of the top *LLS* and partial scores of the rest of the *LLS* by weight factor to alleviate the addition of redundant information.

Inferring links from genomic context: Phylogenetic profile similarity (PG) and Gene neighborhood (GN)

Similar evolutionary conservation pattern between two genes across species are sometimes due to their functional relatedness. This genomic context similarity enables us to infer co-functional links between genes. For constructing RiceNet v2, we used the two most widely used genomic context based network link inferring methods, Phylogenetic profile similarity (PG) (7-9) and gene neighborhood (GN) (10-12). A total of 2,144 sequenced genomes were used. (122 Archae, 1,626 Bacteria and 396 Eukarya genomes)

Phylogenetic profile similarity of two rice genes reflects their co-inheritance during speciation. Co-functionality of genes can be inferred from co-inheritance because genes that function together tend to be inherited together. To measure probability of co-inheritance of two genes, we first ran BLASTp for all *O. sativa* genes against the 2,144 genomes. With the best BLASTp scores for each of genomes, 36,736 (number of *O. sativa* genes) by 2,144 (number of genomes) phylogenetic profile matrix was constructed. The association between two genes based on phylogenetic profiles was measured by mutual information (MI) scores as described in *Date et al.* (13). We did not use the whole concatenated profile of the 2,144 genomes. Rather, sub-profiles for each of three domains of life (Archaea, Bacteria, Eukarya) were separately used which resulted in constructing three networks. These were subsequently integrated to construct a single network. We found that there was substantial increase in the network coverage and accuracy by using this divide-and-integrate approach based on domain-specific phylogenetic profiles.

Two distinct measures of genomic neighborhood exist: i) direct physical distance between neighboring genes (11,12,14), and ii) neighborhood probability (10). There have been evidences that these two measures are complementary (15). We reasoned that if the two methods give complementary information, both of the measurements can be useful. Thus, we inferred co-functional links with both measures. They were subsequently integrated to generate a single GN co-functional network.

Inferring links from literature curated (LC) protein-protein interactions (PPI)

Observing protein-protein interactions (PPIs) in the cell is one of the most popular and certain way to discover the functional associations between genes. To infer the PPI interaction based functional associations for rice, we mined three PPI databases: DIP(16), IntAct (17), MINT (18).

Inferring links from co-expression (CX) patterns

Genes with similar biological functions tend to co-express in diverse biological contexts. High dimensional microarray and RNA-seq data can be used to infer co-functional links between co-expressed genes. We analyzed expression data sets based on four array platforms in GEO (Gene Expression Omnibus) database (19): GPL2025, GPL13160, GPL6864 and GPL8852. To infer co-functional linkages by co-expression patterns, we first created a vector for each gene that contains expression profiles across microarray experiments (GEO samples) in each GEO series. Then we calculated all pairwise Pearson correlation coefficients between vectors to address for co-expression patterns. GEO series with less than 12 samples were not used because measuring correlation with short vectors can generate many promiscuous co-expression patterns between genes. Each GEO series (see **Supplementary table 1**) generated a single co-functional network. Benchmarking with the gold standard set resulted in 39 co-functional networks. They were further integrated into a single CX network for rice.

Links transferred from other species' networks by orthology (Associalogs)

Many biological functions of genes are evolutionarily conserved across species by orthology. This allows transferring the functional information of genes from one species to another. We transferred co-functional linkages from networks of other organisms to RiceNet v2 using the associalog method (20). The links were transferred from three organisms with published genome scale functional gene networks: YeastNet v3 (21) for *Saccharomyces cerevisiae*, WormNet v3 (22) for *Caenorhabtitis elegans*, AraNet v2 (23) for *Arabidopsis thaliana*. In addition, unpublished network links were transferred from three other organisms: *Homo sapiens*, *Danio rerio*, and *Drosophila melanogaster*. Orthology between genes were mapped by using Inparanoid (24).

Supplementary table 1. Comparison between RiceNet v1 and RiceNet v2

Network	**RiceNet v1**	**RiceNet v2**
Gold standard gene pairs for network training	Gene pairs that share the same Gene Ontology biological process terms annotated by TIGR Rice Genome Annotation release 5.	Gene pairs that share pathway terms annotated by at least one of the four databases: i) Biofuel Feedstock Genomics Resource, ii) Kyoto Encyclopedia of Gene and Genomes (KEGG), iii) MapMan, and iv) RiceCyc as of November 2014,
Network size	588,221 links, 19,647 genes	1,775,000 links, 25,765 genes
AT-CX	Associalogs from AraNet v1. Which was constructed by integrating co-expression links from 11 sets comprised of 242 experiments from TAIR	Associalogs from AraNet v2. Which was constructed by integrating co-expression gene links from 64 GEO series comprising 1,261 experiments using the Affymetrix DNA-chip (GPL198) (GSE1491, 2473 , 3350, 4847, 5617, 5620, 5621, 5623, 5624, 5625, 5626, 5627, 5628, 5629, 5630, 5631, 5632, 5633, 5634, 5636, 5637, 5638, 5639, 5686, 5696, 6160, 6176, 7631, 8955, 9674, 9719, 10670, 11262, 12402, 12403, 12887, 13739, 15165, 15689, 16722, 17159, 18071, 18975, 18978, 19520, 19700, 20039, 20454, 21684, 2473, 25067, 26297, 26983, 27985, 30030, 30223, 30492, 31158, 31587, 34667, 35325, 35544, 39384, 42896, 30166
AT-CC	Not Included	Associalogs from AraNet v2 (23)
AT-HT	Not Included	Associalogs from AraNet v2
AT-LC	Associalogs from AraNet v1 (25)	Associalogs from AraNet v2
CE-CC	Associalogs from WormNet v2 (26)	Associalogs from WormNet v3 (22)
CE-CX	Associalogs from WormNet v2	Associalogs from WormNet v3 which was constructed by integrating co-expression links from 12 GEO series of experiments using the Affymetrix DNA-chip (GEO platform GPL200) (GSE11055, 12298, 16050, 19310, 2180, 23528, 25633, 32339, 35354, 6547, 8462, 9682)
DM-CX	Not Included	Associalogs from the co-expression networks of 30 GEO series (GSE2863, 3057, 3854, 5430, 7159, 7614, 7763, 8751, 8892, 10012, 11695, 14517, 14531, 14779, 16152, 16713, 17013, 17874, 21520, 24978, 27163, 27345, 33100, 33779, 33801, 34400, 42255, 46550, 47176, 48997) were integrated.
DM-HT	Not Included	Associalogs from Drosophila high-throughput protein-protein interactions by iRefWeb 4.1 (27)
DR-CX	Not Included	Associalogs from the co-expression networks of 21 GEO series (GSE4201, 8856, 9020, 10188, 11107, 11893, 12991, 13068, 13371, 14495, 14979, 16264, 16740, 17949, 19754, 24528, 32360, 33981, 39731, 47039, 48806) were integrated.

HS-CX	Associalogs from the integrated co-expression network (HS-CX) of HumanNet (28)	Associalogs from the individual co-expression networks of 50 GEO series (GSE2113, 3218, 3307, 5847, 6365, 6477, 6740, 7390, 8052, 8218, 8401, 9419, 9874, 989110327, 10445, 11903, 12662, 13355, 13425, 14034, 14062, 14209, 14323, 14994, 15935, 16015, 16131, 16214, 16476, 17356, 17700, 17855, 17967, 18723, 19577, 20910, 21122, 24427, 26366, 26713, 27155, 28497, 29354, 34211, 34620, 34733, 36701, 39411,) were integrated
HS-HT	Integrated associalogs from affinity purification mass spectrometry analysis data (HS-MS) and high throughput yeast two hybrid data (HS-YH)	Integrated associalogs of high-throughput protein-protein interactions from 6 published literatures. (29-34)
HS-LC	Associalogs from five protein-protein interaction databases (HPRD (35), BIND (36), BioGRID (37), IntAct, MINT)	Associalogs from an integrated protein-protein interactions by HPRD, BioGrid, IntAct, MINT, DIP, iRefWeb data
OS-CX	Integrated individual co-expression networks from 11 GEO series with 274 experiments (GSE4409, 4438,6893, 6901, 7071, 7531, 7532, 7951, 10373, 11157, 16793)	Integrated individual co-expression networks from the 39 GEO series comprising 1345 experiments (GSE6719, 7951, 10373, 11025, 12069, 13988, 14298, 14299, 18361, 18685, 21396, 21397, 21398, 25647, 30136, 30583, 30941, 31077, 31834, 36042, 36043, 36271, 36777, 39426, 39427, 39429, 39635, 39687, 40964, 41556, 41798, 43780, 45571, 48500, 51289, 53417, 54724, 57645, 63110)
OS-GN	Used a probability-based method only	Integration of distance-based and probability-based methods
OS-LC	Not included	Protein-protein interactions from DIP, MINT, IntAct
OS-PG	Inferred co-functional linkages from the phylogenetic profiles of 424 bacterial genomes	Integrated co-functional linkages generated from the profiles of 2144 genomes.(122 Archaea, 396 Eukaryote and 1626 Bacterial genomes)
SC-CC	Associalogs from SC-CC of YeastNet v2 (6)	Associalogs from SC-CC of YeastNet v3 (21)
SC-CX	Associalogs from the integrated co-expression network (SC-CX) of YeastNet v2	Associalogs from the individual co-expression networks from 6 Stanford microarray database sets (Cell cycle, DNA damage, Diauxic, Nutrition, Osmotic stress, and YPD stationary growth) and 40 GEO series (GSE7645, 8799, 9320, 10031, 12220, 12221, 12442, 13684, 14748, 15254, 15936, 16799, 1693, 17364, 17877, 19213, 1934, 20108, 22269, 22832, 23012, 23204, 24802, 24888, 25582, 26829, 26923, 27062, 27235, 30052, 30054, 3076, 31774, 32974, 33276, 33427, 34964, 38848, 40399, 40817) were integrated
SC-GT	Associalogs from the SC-GT of YeastNet v2	Associalogs from the SC-GT of YeastNet v3
SC-HT	Associalogs from links by affinity purification mass spectrometry analysis data (represented as SC-MS in YeastNet v2).	Associalogs from the SC-HT of YeastNet v3
SC-LC	Associalogs from the SC-LC of YeastNet v2	Associalogs from the SC-LC of YeastNet v3

AT-DC<br>CE-GT<br>CE-LC<br>CE-HT<br>CE-GT<br>HS-DC<br>SC-DC<br>SC-TS	Included	Excluded<br>The networks show low prediction power when trained with the new gold standard set.

Datasets described above are denoted by XX-YY. XX represents the names of the species: AT*: Arabidopsis thaliana*, CE: *Caenorhabtitis elegans*, DM: *Drosophila melanogaster*, DR: *Danio rerio*, HS: *Homo sapiens*, OS: *Oryza sativa*, SC: *Saccharomyces cerevisiae*. YY represents the type of data used to infer network links: CX: inferred from co-expression pattern of genes, CC: inferred from co-citation of genes across published papers, DC: inferred from protein domain co-occurrence pattern of the genes, GN: inferred from gene neighborhood, GT: inferred from genetic interactions, HT: inferred from high-throughput protein-protein interaction experiments, LC: inferred by curating protein-protein interactions from the literature, PG: inferred by measuring phylogenetic profile similarity, TS: inferred from protein tertiary structure based protein-protein interaction model.

Supplementary table 2. Ignored pathway terms during generation of gold standard gene pairs

Pathway database	Ignored pathway terms
BFGR GO-BP	DNA integration (GO:001574)<br>Protein phosphorylation (GO:0006468)<br>oxidation-reduction process (GO:0055114)<br>RNA-dependent DNA replication (GO:0006278)<br>regulation of transcription DNA template (GO:0006355)<br>transmembrane transport (GO:0055085)<br>translation (GO:0006412)<br>carbohydrate metabolic process (GO:0005975)<br>proteolysis (GO:0006508)<br>transport (GO:0006810)
KEGG	dosa01100-Metabolic pathways<br>dosa01110-Biosynthesis of secondary molecules<br>dosa01200-Carbon metabolism<br>dosa01210-2-Oxocarboxylic acid metabolism<br>dosa01212-Fatty acid metabolism<br>dosa01230-Biosynthesis of amino acids<br>dosa01220-Degradation of aromatic compounds
MapMan	29.5.11 protein.degradation.ubiquitin<br>30.2.17 signalling.receptor kinases.DUF 26<br>27.3.99 RNA.regulation of transcription.unclassified<br>20.1.7 stress.biotic.PR-proteins<br>29.2.1 protein.synthesis.ribosomal protein<br>30.2.99 signalling.receptor kinases misc<br>29.4.1 protein.postranslational modification.kinase<br>27.3.25 RNA.regulation of transcription.MYB domain transcription factor family<br>30.2.24 signalling.receptor kinases.S-locus glycoprotein like<br>20.2.1 stress.abiotic.heat
RiceCyc	PWY-2881 cytokinins 7-*N*-glucoside biosynthesis<br>PWY-2901 cytokinins 9-*N*-glucoside biosynthesis<br>PWY-2902 cytokinins-*O*-glucoside biosynthesis

Supplementary references

1. Kawahara, Y., de la Bastide, M., Hamilton, J.P., Kanamori, H., McCombie, W.R., Ouyang, S., Schwartz, D.C., Tanaka, T., Wu, J., Zhou, S. *et al.* (2013) Improvement of the Oryza sativa Nipponbare reference genome using next generation sequence and optical map data. *Rice*, **6**, 4.
2. Childs, K.L., Konganti, K. and Buell, C.R. (2012) The Biofuel Feedstock Genomics Resource: a web-based portal and database to enable functional genomics of plant biofuel feedstock species. *Database (Oxford)*, **2012**, bar061.
3. Kanehisa, M., Goto, S., Sato, Y., Kawashima, M., Furumichi, M. and Tanabe, M. (2014) Data, information, knowledge and principle: back to metabolism in KEGG. *Nucleic Acids Res*, **42**, D199-205.
4. Thimm, O., Blasing, O., Gibon, Y., Nagel, A., Meyer, S., Kruger, P., Selbig, J., Muller, L.A., Rhee, S.Y. and Stitt, M. (2004) MAPMAN: a user-driven tool to display genomics data sets onto diagrams of metabolic pathways and other biological processes. *Plant J*, **37**, 914-939.
5. Jaiswal, P., Ni, J., Yap, I., Ware, D., Spooner, W., Youens-Clark, K., Ren, L., Liang, C., Zhao, W., Ratnapu, K. *et al.* (2006) Gramene: a bird's eye view of cereal genomes. *Nucleic Acids Res*, **34**, D717-723.
6. Lee, I., Li, Z. and Marcotte, E.M. (2007) An improved, bias-reduced probabilistic functional gene network of baker's yeast, Saccharomyces cerevisiae. *PLoS One*, **2**, e988.
7. Huynen, M., Snel, B., Lathe, W., 3rd and Bork, P. (2000) Predicting protein function by genomic context: quantitative evaluation and qualitative inferences. *Genome Res*, **10**, 1204-1210.
8. Pellegrini, M., Marcotte, E.M., Thompson, M.J., Eisenberg, D. and Yeates, T.O. (1999) Assigning protein functions by comparative genome analysis: protein phylogenetic profiles. *Proc Natl Acad Sci U S A*, **96**, 4285-4288.
9. Wolf, Y.I., Rogozin, I.B., Kondrashov, A.S. and Koonin, E.V. (2001) Genome alignment, evolution of prokaryotic genome organization, and prediction of gene function using genomic context. *Genome Res*, **11**, 356-372.
10. Bowers, P.M., Pellegrini, M., Thompson, M.J., Fierro, J., Yeates, T.O. and Eisenberg, D. (2004) Prolinks: a database of protein functional linkages derived from coevolution. *Genome Biol*, **5**, R35.
11. Dandekar, T., Snel, B., Huynen, M. and Bork, P. (1998) Conservation of gene order: a fingerprint of proteins that physically interact. *Trends Biochem Sci*, **23**, 324-328.
12. Overbeek, R., Fonstein, M., D'Souza, M., Pusch, G.D. and Maltsev, N. (1999) The use of gene clusters to infer functional coupling. *Proc Natl Acad Sci U S A*, **96**, 2896-2901.
13. Date, S.V. and Marcotte, E.M. (2003) Discovery of uncharacterized cellular systems by genome-wide analysis of functional linkages. *Nat Biotechnol*, **21**, 1055-1062.
14. Korbel, J.O., Jensen, L.J., von Mering, C. and Bork, P. (2004) Analysis of genomic context: prediction of functional associations from conserved bidirectionally transcribed gene pairs. *Nat Biotechnol*, **22**, 911-917.
15. Shin, J., Lee, T., Kim, H. and Lee, I. (2014) Complementarity between distance- and probability-based methods of gene neighbourhood identification for pathway reconstruction. *Mol Biosyst*, **10**, 24-29.

16. Salwinski, L., Miller, C.S., Smith, A.J., Pettit, F.K., Bowie, J.U. and Eisenberg, D. (2004) The Database of Interacting Proteins: 2004 update. *Nucleic Acids Res*, **32**, D449-451.
17. Orchard, S., Ammari, M., Aranda, B., Breuza, L., Briganti, L., Broackes-Carter, F., Campbell, N.H., Chavali, G., Chen, C., del-Toro, N. *et al.* (2014) The MIntAct project--IntAct as a common curation platform for 11 molecular interaction databases. *Nucleic Acids Res*, **42**, D358-363.
18. Licata, L., Briganti, L., Peluso, D., Perfetto, L., Iannuccelli, M., Galeota, E., Sacco, F., Palma, A., Nardozza, A.P., Santonico, E. *et al.* (2012) MINT, the molecular interaction database: 2012 update. *Nucleic Acids Res*, **40**, D857-861.
19. Barrett, T., Wilhite, S.E., Ledoux, P., Evangelista, C., Kim, I.F., Tomashevsky, M., Marshall, K.A., Phillippy, K.H., Sherman, P.M., Holko, M. *et al.* (2013) NCBI GEO: archive for functional genomics data sets--update. *Nucleic Acids Res*, **41**, D991-995.
20. Kim, E., Kim, H. and Lee, I. (2013) JiffyNet: a web-based instant protein network modeler for newly sequenced species. *Nucleic Acids Res*, **41**, W192-197.
21. Kim, H., Shin, J., Kim, E., Kim, H., Hwang, S., Shim, J.E. and Lee, I. (2014) YeastNet v3: a public database of data-specific and integrated functional gene networks for Saccharomyces cerevisiae. *Nucleic Acids Res*, **42**, D731-736.
22. Cho, A., Shin, J., Hwang, S., Kim, C., Shim, H., Kim, H., Kim, H. and Lee, I. (2014) WormNet v3: a network-assisted hypothesis-generating server for Caenorhabditis elegans. *Nucleic Acids Res*, **42**, W76-82.
23. Lee, T., Yang, S., Kim, E., Ko, Y., Hwang, S., Shin, J., Shim, J.E., Shim, H., Kim, H., Kim, C. *et al.* (2015) AraNet v2: an improved database of co-functional gene networks for the study of Arabidopsis thaliana and 27 other nonmodel plant species. *Nucleic Acids Res*, **43**, D996-D1002.
24. Sonnhammer, E.L. and Ostlund, G. (2015) InParanoid 8: orthology analysis between 273 proteomes, mostly eukaryotic. *Nucleic Acids Res*, **43**, D234-239.
25. Lee, I., Ambaru, B., Thakkar, P., Marcotte, E.M. and Rhee, S.Y. (2010) Rational association of genes with traits using a genome-scale gene network for Arabidopsis thaliana. *Nat Biotechnol*, **28**, 149-156.
26. Lee, I., Lehner, B., Vavouri, T., Shin, J., Fraser, A.G. and Marcotte, E.M. (2010) Predicting genetic modifier loci using functional gene networks. *Genome Res*, **20**, 1143-1153.
27. Turinsky, A.L., Razick, S., Turner, B., Donaldson, I.M. and Wodak, S.J. (2014) Navigating the global protein-protein interaction landscape using iRefWeb. *Methods Mol Biol*, **1091**, 315-331.
28. Lee, I., Blom, U.M., Wang, P.I., Shim, J.E. and Marcotte, E.M. (2011) Prioritizing candidate disease genes by network-based boosting of genome-wide association data. *Genome Res*, **21**, 1109-1121.
29. Ewing, R.M., Chu, P., Elisma, F., Li, H., Taylor, P., Climie, S., McBroom-Cerajewski, L., Robinson, M.D., O'Connor, L., Li, M. *et al.* (2007) Large-scale mapping of human protein-protein interactions by mass spectrometry. *Mol Syst Biol*, **3**, 89.
30. Havugimana, P.C., Hart, G.T., Nepusz, T., Yang, H., Turinsky, A.L., Li, Z., Wang, P.I., Boutz, D.R., Fong, V., Phanse, S. *et al.* (2012) A census of human soluble protein complexes. *Cell*, **150**, 1068-1081.

31. Hutchins, J.R., Toyoda, Y., Hegemann, B., Poser, I., Heriche, J.K., Sykora, M.M., Augsburg, M., Hudecz, O., Buschhorn, B.A., Bulkescher, J. *et al.* (2010) Systematic analysis of human protein complexes identifies chromosome segregation proteins. *Science*, **328**, 593-599.
32. Sowa, M.E., Bennett, E.J., Gygi, S.P. and Harper, J.W. (2009) Defining the human deubiquitinating enzyme interaction landscape. *Cell*, **138**, 389-403.
33. Wang, J., Huo, K., Ma, L., Tang, L., Li, D., Huang, X., Yuan, Y., Li, C., Wang, W., Guan, W. *et al.* (2011) Toward an understanding of the protein interaction network of the human liver. *Mol Syst Biol*, **7**, 536.
34. Yu, H., Tardivo, L., Tam, S., Weiner, E., Gebreab, F., Fan, C., Svrzikapa, N., Hirozane-Kishikawa, T., Rietman, E., Yang, X. *et al.* (2011) Next-generation sequencing to generate interactome datasets. *Nat Methods*, **8**, 478-480.
35. Keshava Prasad, T.S., Goel, R., Kandasamy, K., Keerthikumar, S., Kumar, S., Mathivanan, S., Telikicherla, D., Raju, R., Shafreen, B., Venugopal, A. *et al.* (2009) Human Protein Reference Database--2009 update. *Nucleic Acids Res*, **37**, D767-772.
36. Galperin, M.Y. (2008) The Molecular Biology Database Collection: 2008 update. *Nucleic Acids Res*, **36**, D2-4.
37. Chatr-Aryamontri, A., Breitkreutz, B.J., Oughtred, R., Boucher, L., Heinicke, S., Chen, D., Stark, C., Breitkreutz, A., Kolas, N., O'Donnell, L. *et al.* (2015) The BioGRID interaction database: 2015 update. *Nucleic Acids Res*, **43**, D470-478.
